# Optimal control analysis of Ebola disease with control strategies of quarantine and vaccination

**DOI:** 10.1186/s40249-016-0161-6

**Published:** 2016-07-13

**Authors:** Muhammad Dure Ahmad, Muhammad Usman, Adnan Khan, Mudassar Imran

**Affiliations:** Department of Mathematics, Prince Sultan University, Riyadh, Kingdom of Saudi Arabia; Department of Mathematics, University of Dayton, Dayton, OH USA; Department of Mathematics, Lahore University of Management Sciences, Lahore, Pakistan; Department of Mathematics and Natural Sciences, GULF University for Science and Technology, Mishref, Kuwait

**Keywords:** Epidemiology, Ebola virus disease, Optimal control strategies, Disease transmission

## Abstract

**Background:**

The 2014 Ebola epidemic is the largest in history, affecting multiple countries in West Africa. Some isolated cases were also observed in other regions of the world.

**Method:**

In this paper, we introduce a deterministic SEIR type model with additional hospitalization, quarantine and vaccination components in order to understand the disease dynamics. Optimal control strategies, both in the case of hospitalization (with and without quarantine) and vaccination are used to predict the possible future outcome in terms of resource utilization for disease control and the effectiveness of vaccination on sick populations. Further, with the help of uncertainty and sensitivity analysis we also have identified the most sensitive parameters which effectively contribute to change the disease dynamics. We have performed mathematical analysis with numerical simulations and optimal control strategies on Ebola virus models.

**Results:**

We used dynamical system tools with numerical simulations and optimal control strategies on our Ebola virus models. The original model, which allowed transmission of Ebola virus via human contact, was extended to include imperfect vaccination and quarantine. After the qualitative analysis of all three forms of Ebola model, numerical techniques, using MATLAB as a platform, were formulated and analyzed in detail. Our simulation results support the claims made in the qualitative section.

**Conclusion:**

Our model incorporates an important component of individuals with high risk level with exposure to disease, such as front line health care workers, family members of EVD patients and Individuals involved in burial of deceased EVD patients, rather than the general population in the affected areas. Our analysis suggests that in order for *R*_0_ (i.e., the basic reproduction number) to be less than one, which is the basic requirement for the disease elimination, the transmission rate of isolated individuals should be less than one-fourth of that for non-isolated ones. Our analysis also predicts, we need high levels of medication and hospitalization at the beginning of an epidemic. Further, optimal control analysis of the model suggests the control strategies that may be adopted by public health authorities in order to reduce the impact of epidemics like Ebola.

**Electronic supplementary material:**

The online version of this article (doi:10.1186/s40249-016-0161-6) contains supplementary material, which is available to authorized users.

## Multilingual abstracts

Please see Additional file 1 for translations of the abstract into the six official working languages of the United Nations.

## Background

Ebola hemorrhagic fever, commonly known as Ebola virus disease (EVD), is presumed to have started during the mid seventies in Guinea. As a result of this outbreak, 3000 cases and 1500 deaths were confirmed in Guinea, Liberia, Nigeria and Sierra Leone. During the period, two separate outbreaks involving 284 cases (with 280 deaths) centered in Nzara, Sudan and 318 cases (280 deaths) in Yambuka (near the Ebola river), Democratic Republic of Congo (formally known as Zaire) were also reported. Since these original cases, there were approximately 20 major outbreaks through the year 2013 [24]. The recent outbreak, which began in Guinea during early 2014 and later on spread to Liberia and Sierra Leone, is the longest, largest and most widespread Ebola outbreak and therefore on August 08, 2014, World Health Organization (WHO) declared the epidemic to be a Public Health Emergency of International Concern (PHEIC) [23]. According to the WHO report, as of November 2nd, 2014, a total of 13042 suspected cases and 4818 confirmed deaths were recorded. Among these deaths, 4791 (approximately 99.5 %) occurred in west African countries [4].

The causative agent of Ebola virus is an RNA virus of family Filoviridae and genus Ebola virus which includes 3 genera: Cueva-virus, Marburg-virus, and Ebola virus. So far, five species of Ebola virus strains have been identified, named as Zaire, Bundibugyo, Sudan, Reston and Tai Forest. The recent wide spread outbreak of EVD in western Africa was due to the first three, Bundibugyo Ebola virus, Zaire Ebola virus, and Sudan Ebola virus, and in particular, the current outbreak of 2014 belongs to the Zaire species [7, 11].

Ebola virus is generally introduced into the human population through close contact with the blood, secretions, organs or other bodily fluids of infected animals such as chimpanzees, gorillas, fruit bats, monkeys, forest antelope and porcupines found ill or dead or in the rainforest. Later on human to human transmission can occur only via direct contact with the blood or other bodily fluid from an infected person as well as by contact with objects recently contaminated by an actively ill infected individual [11]. Since the people remain infectious as long as their blood and body fluids contain the virus, burial ceremonies in which mourners have direct contact with the body of the deceased person can also play a role in the transmission of disease.

The Ebola incubation period can be as short as 2 days and as long as 21 days [13]. On average the symptoms of Ebola, such as, fever fatigue, muscular pain, vomiting and diarrhoea, can appear within four to six days after a person becomes infected with the Ebola virus. Recent results showed a promising breakthrough in a vaccination, [8], but we are still far behind its availability and usage for the common people. However in the absence of treatment through vaccination, these specific early symptoms of EVD and supportive care-rehydration with oral or intravenous fluids can improve the survival rate. In general, the transmission of the disease is less likely to occur during the incubation period and the transmissibility increases with the duration of disease and direct contact with the patients during the late stage of illness.

The objective of this paper is to understand the Ebola disease dynamics through the lens of Mathematical modeling and to predict the possible future outcome in terms of resource utilization for the disease control and the effectiveness of the future vaccination on sick population. Since economic resources are limited, epidemiological models have started taking into consideration the economic constraints imposed by limited resources when analyzing control strategies. The successful eradication of the emerging diseases, like Ebola, not only depends on the availability of medical infrastructures but also on the ability to understand the transmission dynamics of the disease as well as the application of optimal control strategies and the implementation of logistic policies. Therefore, in this paper, we presented a mathematical model to address these issues and discuss various optimal strategies for detection, prevention and control of the disease.

During recent years, a variety of computational and statistical models have been documented in literature to characterize and resolve the mechanisms underlying the trends in the growth of EVD, see for instance [3, 9, 14] and [15]. The models, we presented in this paper explain different aspects of the disease dynamics. Our model is based on the SEIR type formulation, along with an extended susceptible class (*S*_*L*_- low risk susceptibles and *S*_*H*_-high risk susceptibles) and an additional class of hospitalized patients. We used heuristic arguments that was developed on the foundation of deterministic approach rather than stochastic one to discuss the disease control strategies. The reasons were very obvious, as the material properties of the model variables and parameters are well known, (i.e. deterministic) and the applied control measures (like isolation, quarantine etc) are also well established. Further, this deterministic approach is sufficient to model the dynamics of different population groups in the case where the infected population is much smaller in size as compared to the total population (as in the case of Ebola). Finally, as a matter of fact, the expected value of the stochastic model and the deterministic models possess a similar asymptotic behaviour in this case. To analyze and understand the EVD transmission dynamics, we perform stability and steady state analysis and also calculated the effective reproduction number *R*_0_, that measures the average number of secondary cases generated by a typical primary case at a given time. The numerical value of *R*_0_, based on the parameter values documented in literature, helps us to assess the current status of the evolving epidemic outbreak and upward (when *R*_0_>1) or downward (*R*_0_<1) trend of the disease.

In the second part, we introduce a new quarantine class along with hospitalization in order to assess the impact of quarantine and isolation in combatting the spread of diseases. We define the quarantine as the removal of individuals suspected of being exposed to EVD from the general population. We perform optimal control analysis to predict and suggest the optimal strategies to overcome the epidemic in this scenario. In the last section, we include a vaccination class and again use the optimal control method to suggest how minimal resources can be used to eradicate the disease.

## Method

**Fig. 1:**
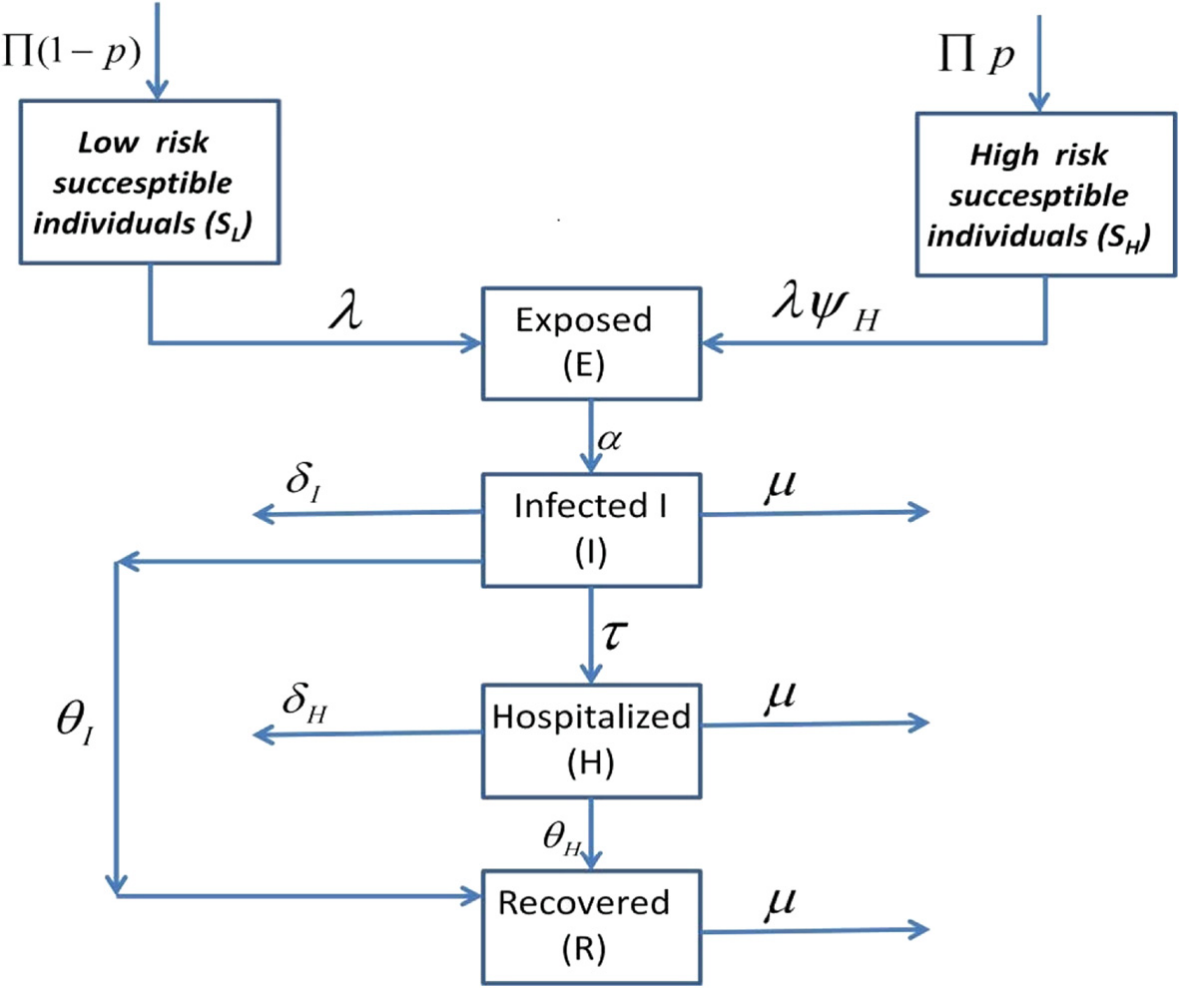
Flow diagram of the main model

Our proposed epidemic model of the Ebola virus disease is similar to our previous model discussed in [1]. The total population is divided into six mutually exclusive compartments which are classified as, low risk susceptible (*S*_*L*_), high risk susceptible (*S*_*H*_), exposed (*E*), infected (*I*), hospitalized (*H*) and recovered (*R*). High risk susceptible are individuals having high rate of acquiring infection *ψ*_*H*_>1. Usually high risk includes the women, children, health care units and doctors. The rest of the population is included in the low risk susceptible section. Also all newborns are assumed to be low risk susceptible as there is no vertical transmission of the infection. The flow diagram depicted in Fig. 1 explains the procedure of model formulation and our complete model describing the Ebola dynamics is given below.

1 $$\begin{array}{@{}rcl@{}} \frac{dS_{L}}{dt}&=&\Pi(1-p)-\lambda S_{L}-\mu S_{L}  \\ \frac{dS_{H}}{dt}&=&\Pi p-\psi_{_{H}}\lambda S_{H} -\mu S_{H}, \quad \psi_{_{H}}>1  \\ \frac{dE}{dt}&=& \lambda (S_{L}+ \psi_{_{H}} S_{H})-(\alpha+\mu)E \\ \frac{dI}{dt}&=&\alpha E - (\tau +\theta_{I} +\delta_{I} +\mu)I  \\ \frac{dH}{dt} &=& \tau I -(\theta_{H} +\delta_{H} +\mu)H  \\ \frac{dR}{dt} &=& \theta_{I} I +\theta_{H} H -\mu R  \end{array} $$

where

$$ \lambda=\frac{\beta(I+\eta H)}{N} $$

The variables and parameters used in our model along with their description and values used in the manuscript are listed in Tables 1 and 2. The parameter values we used have been well documented in the literature, see for instance [6, 9, 18] and [1]. Further, it is common practice to use the case definition of EVD hospitalization in public, therefore we use the same values of hospitalization rate.

**Table 1:** Description of the variables of the model

Variable	Description
*N*_*H*_	Total human population
*S*_*H*_	Population of high risk susceptible individuals
*S*_*L*_	Population of low risk susceptible individuals
*E*	Population of exposed individuals
*I*	Population of infected individuals
*H*	Population of hospitalized individuals
*R*	Population of recovered individuals

**Table 2:** Description of the parameters of the model

Parameter	Description	Value
*Π*	Recruitment rate	1.7
1/*μ*	Average life of human	63
*β*	Transmission rate of disease	0.20–0.39
*ψ*_*H*_	Modification parameter for infection rate	
	of high risk susceptible individuals	1.2–2
*δ*_*I*_	Disease-induced death rate of infected individuals	0.10
*δ*_*H*_	Disease-induced death rate of hospitalized	
	individuals	0.5
*θ*_*I*_	Recovery rate of infected individuals	0.10
*θ*_*H*_	Recovery rate of hospitalized individuals	0.20
*α*	Rate at which latent individuals become infectious	0.10
*τ*	Hospitalization rate for infected individuals	0.16
*η*	Modification parameter for relative infectiousness	
	of hospitalized individuals	1.0–1.5

### Stability of equilibria and Reproduction Number

*Disease Free equilibrium:* It can be easily seen that our model-1 satisfies all the conditions of the positivity and boundness theorem (see Theorem A-4 of [12]), so the solution of the model is unique and is bounded for all non-negative time. Further the disease free equilibrium is given by

$$\begin{array}{@{}rcl@{}} \mathcal{E}_{0}&=(S_{L}^{*},S_{H}^{*},E^{*},I^{*},H^{*},R^{*})=\left(S_{L}^{*},S_{H}^{*},0,0,0,0,\right), \end{array} $$

with,

$$S_{L}^{*}=\frac{\Pi(1-p)}{\mu}~ \text{and}~ S_{H}^{*}=\frac{\Pi{p}}{\mu}. $$

Following [5], the linear stability of $\mathcal {E}_{0}$ can be established using the next generation operator method on system-(1).

*Reproduction number:* The basic reproduction number *R*_0_ is the number of individuals infected by a single infected individual during the infectious period in the entire susceptible population. By using the next generation matrix approach [1, 20, 22], the expression of *R*_0_ leads to

2 $$ \begin{aligned} R_{0}&= \alpha\beta\Omega\! \left\{\!\! \frac{1}{(\alpha \!+ \!\mu)(\theta_{I}\,+\,\delta_{I}\,+\,\mu)\,+\,\tau (\alpha \,+\,\mu)} \,+\, \frac{\tau}{(\alpha \,+\,\mu)(\theta_{I}\,+\,\delta_{I}\,+\,\mu)\,+\,\tau (\alpha\,+\,\mu)}\frac{\eta}{K_{3}}\! \right\}\!\!,\\ &= R_{1}+R_{2} \end{aligned}  $$

Here *R*_1_ denotes strands for the continuation of infectious individuals from the community and *R*_2_ represents the strand from the hospital community. The epidemiological significance of the *reproduction number*, *R*_0_, which represents the average number of new cases generated by a primary infectious individual in a population where some susceptible individuals are at high risk, and some infected individuals go to hospital, is that the Ebola pandemic can be effectively controlled by reducing the number of high risk individuals and by decreasing the contacts of hospitalized individuals with other individuals that may include relatives and health care workers. This can bring the threshold quantity (*R*_0_) to a value less than unity. Biologically, the following Lemma implies that the Ebola pandemic can be eliminated from the population (when *R*_0_<1) if the initial sizes of the sub-populations in various compartments of the model are in the basin of attraction of the DFE $(\mathcal {E}_{0})$.

#### **Lemma**.

**Fig. 2:**
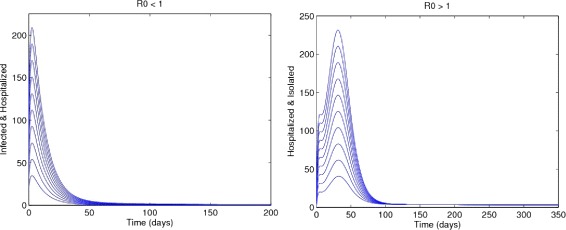
Time series Graphs for *R*_0_<1 (*Left Panel*) and *R*_0_>1 (*Right Panel*)

The DFE, $\mathcal {E}_{0},$ of the model 1, is locally asymptotically stable (LAS) if *R*_0_<1, and unstable if *R*_0_>1. Figure 2 clearly depict the results of the above lemma.

*Endemic Equilibrium:* Let $(S_{L}^{*},S_{H}^{*},E^{*},I^{*},R^{*})$ represents any arbitrary endemic equilibrium of the model-(1), such that $N^{*}= S_{L}^{*}+S_{H}^{*}+E^{*}+I^{*}+R^{*}$. Their values are obtained by solving our system and are given by

3 $$\begin{array}{@{}rcl@{}} E^{*} &=& \frac{\lambda^{*}(S_{L}^{*}+\psi_{H}^{*} S_{H}^{*})}{k_{1}} \end{array} $$

4 $$\begin{array}{@{}rcl@{}} I^{*} &=& \frac{\alpha \lambda^{*}(S_{L}^{*} +\psi_{H}^{*} S_{H}^{*}}{k_{1} k_{2}} \end{array} $$

5 $$\begin{array}{@{}rcl@{}} H^{*} &=& \frac {\alpha \tau}{k_{1} k_{2} k_{3}} \lambda^{*} (S_{L}^{*}+\psi_{H}^{*}S_{H}^{*}) \end{array} $$

6 $$\begin{array}{@{}rcl@{}} R^{*} &=& P_{1} \lambda^{*}(S_{L}^{*}+\psi_{H}^{*}S_{H}^{*}) \end{array} $$

where *k*_1_=*μ*+*α*, *k*_2_=*τ*+*θ*_*I*_+*δ*_*I*_+*μ* & *k*_3_=*μ*+*θ*_*H*_+*δ*_*H*_, and

$$P_{1} = \left[\frac{\theta_{I} \alpha}{k_{1} k_{2}} + \frac{\theta_{H} \tau \alpha}{k_{1} k_{2} k_{3}}\right]\frac{1}{\mu} $$

Now substituting these expressions in $\lambda ^{*} = \frac {\beta (I^{*}+\eta H^{*})}{N^{*}}$, we get

$$\begin{aligned} \lambda^{*2}&\left[(X+Y+Z+P_{1})(S_{L}^{*}+\psi^{*}S_{H}^{*})\right] -\lambda^{*}\\ &\left[\beta (X+\eta Y)(S_{L}^{*}+\psi^{*}S_{H}^{*}) + S_{L}^{*}\right] +S_{H}^{*}=0, \end{aligned} $$

where

$$X = \frac{\alpha}{k_{1} k_{2}}, \quad \quad Y=\frac{\tau \alpha}{k_{1} k_{2} k_{3}}, \quad and \quad Z=\frac{1}{k_{1}}. $$

It is clear from the last equation that *λ*^∗2^ can have at most two solutions, depending on the values of parameter used.

### Uncertainty and sensitivity analysis

The prevalence of a disease in any population can be determined by the threshold quantity, basic reproduction number *R*_0_, as given by equation (2). Since our model is deterministic in nature, the only sources of uncertainty are the model parameters and the initial conditions. The basic reproductive ratio *R*_0_ would usually be estimated by the input of specific parameter values; however, factors such as natural variation, errors in measurements and lack of measuring techniques contribute towards the associated uncertainty of the model parameters. In such a scenario, it is more appropriate to treat each parameter as a random variable, distributed according to an appropriate probability distribution. We use Latin Hypercube sampling, independently from each of the parameter distributions. These samples are then randomly permuted to form the input parameter vectors. These samples are then used to calculate the distribution for values of *R*_0_ [1].

In general, uncertainty analysis quantifies the degree of confidence in the parameter estimates by producing 95 % confidence intervals (CI) which can be interpreted as intervals containing 95 % of future estimates when the same assumptions are made and the only noise source is observation error. Additionally, sensitivity analysis identifies critical model parameters and quantifies the impact of each input parameter on the value of an output. In this section, we shall perform uncertainty and sensitivity analysis of the basic reproduction number *R*_0_. A detailed description of the history and methodology of uncertainty and sensitivity analysis is given in [16].

**Fig. 3:**
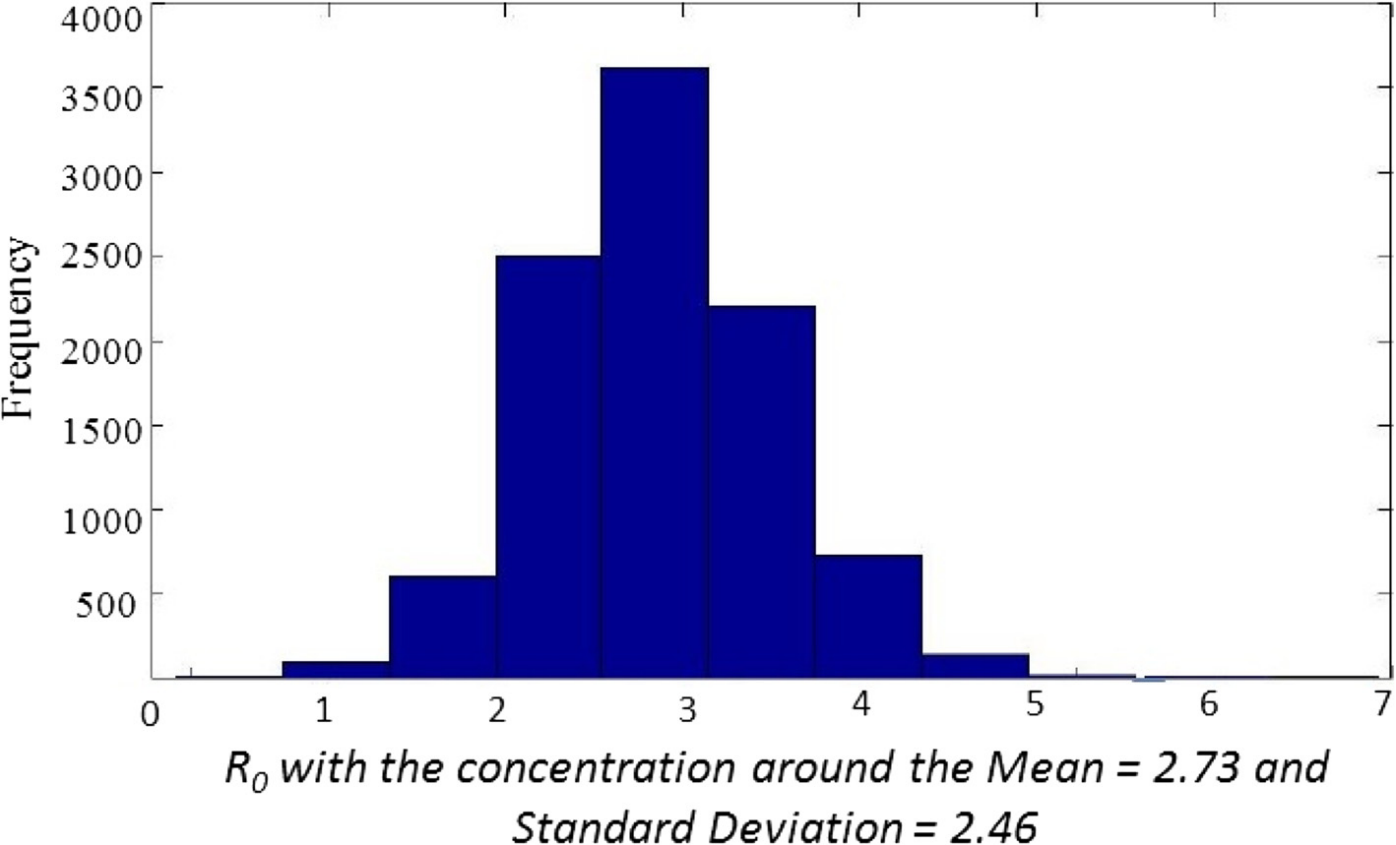
Uncertainty analysis of *R*_0_

**Fig. 4:**
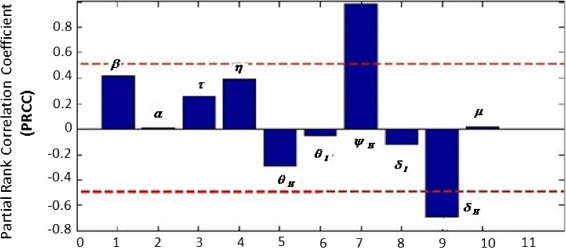
Sensitivity analysis of the basic reproduction number *R*_0_

Since we lack any concrete information about probability distributions of the model parameters, we assume that our model parameters are normally distributed although it is quite possible that some parameters are skewed towards a particular value. The parameters used for uncertainty and sensitivity analysis are given in Table 3, and we assume that the recruitment rate *Π* is constant. Results of uncertainty and sensitivity analysis are presented in Figs. 3 and 4.

**Table 3:** Parameter values used in uncertainty and sensitivity analysis

Parameter	Mean value	Standard deviation
*β*	0.88	0.2
*η*	0.5	0.200595
*δ*_*H*_	0.8	0.595029
*τ*	0.8	0.0999323
*θ*_*H*_	2.0	0.19709
*α*	0.2	0.142107
*θ*_*I*_	0.3	0.0149564
*δ*_*I*_	0.5	0.050117
*ψ*_*H*_	0.99	1.9891
*μ*	0.00003	1.99×10^−6^

Uncertainty analysis yields 95 % confidence interval for the value of the basic reproduction number *R*_0_ to be (2.73, 2.97) with the standard deviation of 2.46.

Sensitivity of values of *R*_0_ to the uncertainty in the parameter values is assessed next; we identify crucial model parameters by computing partial rank correlation coefficient (PRCC) which is a measured impact of each input parameter on the output, i.e., *R*_0_. PRCC reduces the non-linearity effects by rearranging the data in ascending order, replacing the values with their ranks and then providing the measure of monotonicity after the removal of the linear effects of each model parameter keeping all other parameters constant [16]. The horizontal lines in Fig. 4 represent the significant range of correlation, i.e., |*PRCC*|>0.5. The sensitivity analysis suggests that the most significant parameters are *ψ*_*H*_ and *δ*_*H*_ and hence, these parameters should be estimated with precision to accurately capture the dynamics of the infection.

## Optimal control analysis

Pontryagin and Boltyanskii [19] formulated optimal control theory for models with underlying dynamics defined by a system of ordinary differential equations. Since its discovery, this algorithm has progressed considerably. In particular, recently their procedure has been employed widely to make decisions involving epidemic and biological models. See for instance [2, 10, 17]. The basis of this theory is to find a control law for a given system which undergoes the aforementioned optimality criterion. The Pontryagin’s Maximum Principle allows us to adjust the control in a model to achieve the desired results. The control parameters are mostly functions of time, mainly appearing as the coefficients in the model. A typical problem includes a cost functional consisting of control and state variables and the aim is to minimize the cost functional.

In this paper, we will use Pontryagin’s maximum principle on two variations of our original model to optimize the cost and resources. In the first case, we modified our model for the case of Hospitalization and Quarantine, while in the second case, we perform optimal control strategies on Hospitalization and Vaccination. These modifications in our models along with the mathematical formulation of the optimal control procedure are explained in the last section of the paper. In this paper, we have worked on the analytical side of the optimal theory as well as we used numerical techniques to supported our claims. These results are discussed in the following sections.

### Case 1: hospitalization and quarantine

Since Ebola virus disease is very severe, a common way to control the spread of the disease is to employ isolation/quarantine. In order to introduce this clause in our model, the original model described above is modified and exposed individuals are now quarantined at a rate *ξ*. Further we assume that these quarantined individuals are also hospitalized at a rate *τ*_*Q*_. Quarantined individuals can also die at a natural death rate *μ*. Therefore by using these assumptions the updated model is given as

7 $$\begin{array}{@{}rcl@{}} \frac{dS_{L}}{dt}&=\Pi(1-p)-\lambda S_{L}-\mu S_{L}  \\ \frac{dS_{H}}{dt}&=\Pi p-\psi_{_{H}}\lambda S_{H} -\mu S_{H}  \\ \frac{dE}{dt}&= \lambda (S_{L}+ \psi_{_{H}} S_{H})-(\alpha+\mu)E-\xi E  \\ \frac{dQ}{dt}&= \xi E -(\tau_{_{Q}} +\mu)Q \\ \frac{dI}{dt}&= \alpha E - (\tau +\theta_{I} +\delta_{I} +\mu)I  \\ \frac{dH}{dt} &= \tau I +\tau_{_{Q}}Q -(\theta_{H} +\delta_{H} +\mu)H  \\ \frac{dR}{dt} &= \theta_{I} I +\theta_{H} H-\mu R  \end{array} $$

where

$$ \lambda=\frac{\beta(I+\eta H)}{N}. $$

To find an optimal hospitalization and quarantine strategy to control Ebola, we assume that the control set is given as

8 $$ \begin{aligned} \mathbf{U} = \left[\xi (t), \tau_{Q}(t), \tau(t): 0\le\xi (t), \tau_{Q}(t), \tau (t)\le \zeta_{i},\right.\\ \left.0\le t \le T, 0< \zeta_{i} \le 1, (i=1, 2, 3)\right]. \end{aligned}  $$

Our aim is to minimize the cost function given as:

9 $${} \begin{aligned} J[\xi, \tau_{Q}, &\tau]= {\int_{0}^{T}}\\ & \left[I +Q+ \frac{1}{2}W_{1}\xi^{2}+\frac{1}{2} W_{2}{\tau_{Q}^{2}}(t)+\frac{1}{2} W_{3}\tau^{2}(t)\right] dt. \end{aligned}  $$

Our objective is to find an optimal control for the quarantine rate *ξ*^∗^(*t*), and hospitalization rates $\tau _{Q}^{*}(t), \tau ^{*}(t)$ such that $J[\xi ^{*}, \tau _{Q}^{*}, \tau ^{*}]= {min}_{_{({\xi, \tau _{Q}, \tau \in U})}} J[\xi, \tau _{Q}, \tau ]$.

### Case 2: hospitalization and vaccination

We assume that there exists some vaccination to prevent individuals from the Ebola virus disease. To take account of the vaccination effect, low and high risk susceptible are vaccinated at the rate of *γ*_1_ and *γ*_2_ respectively. Hence the updated model is given as

10 $$\begin{array}{@{}rcl@{}} \frac{dS_{L}}{dt}&=\Pi(1-p)-\lambda S_{L}-\gamma_{1} S_{L}-\mu S_{L} \\ \frac{dS_{H}}{dt}&=\Pi p-\psi_{_{H}}\lambda S_{H} -\gamma_{2} S_{H}-\mu S_{H}  \\ \frac{dV}{dt}&=\gamma_{1} S_{L} +\gamma_{2} S_{H} -\lambda(1-\epsilon)V-\mu V  \\ \frac{dE}{dt}&= \lambda (S_{L}+ \psi_{_{H}} S_{H})+\lambda(1-\epsilon)V-(\alpha+\mu)E\\ \frac{dI}{dt}&= \alpha E - (\tau +\theta_{I} +\delta_{I} +\mu)I  \\ \frac{dH}{dt} &= \tau I -(\theta_{H} +\delta_{H} +\mu)H  \\ \frac{dR}{dt} &= \theta_{I} I +\theta_{H} H-\mu R  \end{array} $$

where

$$ \lambda=\frac{\beta(I+\eta H)}{N}. $$

To find an optimal hospitalization and vaccination strategy to control Ebola, let the control set is given as

11 $$ \begin{aligned} \mathbf{U} = \left[\gamma_{1} (t), \gamma_{2} (t), \tau(t): 0\le\gamma_{1} (t), \gamma_{2} (t), \tau (t)\le \zeta_{i},\right. \\ \left.0\le t \le T, 0< \zeta_{i} \le 1, (i=1, 2, 3)\right]. \end{aligned}  $$

Our aim is to minimize the cost function given as:

12 $$ \begin{aligned} J[\gamma_{1}, \gamma_{2}, \tau]= {{\int_{0}^{T}}{\left[ I+ \frac{1}{2} W_{1}{\gamma_{1}^{2}}+\frac{1}{2} W_{2}{\gamma_{2}^{2}}(t)+\frac{1}{2} W_{3}\tau^{2}(t)\right] dt}}. \end{aligned}  $$

Similar to the quarantine case, again our objective is to find an optimal control for vaccination rates $\gamma _{1}^{*}(t),\gamma _{2}^{*} (t)$, and hospitalization rate *τ*^∗^(*t*) such that $J[\gamma _{1}^{*}, \gamma _{2}^{*}, \tau ^{*}]= {min}_{_{{\gamma _{1}, \gamma _{2}, \tau \in U}}} J[\gamma _{1}, \gamma _{2}, \tau ]$.

The formulation of the Hamiltonian and its adjoint system along with the procedure of finding the optimality conditions for these two cases are discussed in the Appendix.

### Simulation results

In this section, numerical solutions of the given systems along with the adjoint systems of both of the cases (Hospitalization and Quarantine, and Hospitalization and Vaccination) are provided. The system is integrated using the Runge-Kutta method using the Matlab platform. Further the value of *J*, the cost functional that had to be minimized, is calculated numerically and graphed (again for the hospitalization and quarantine section). Further, simulations of the population of infected individuals from optimal control and constant control are compared. Finally, the optimal control solutions of the control variables are provided for several values of *β*.

#### Hospitalization and quarantine

**Fig. 5:**
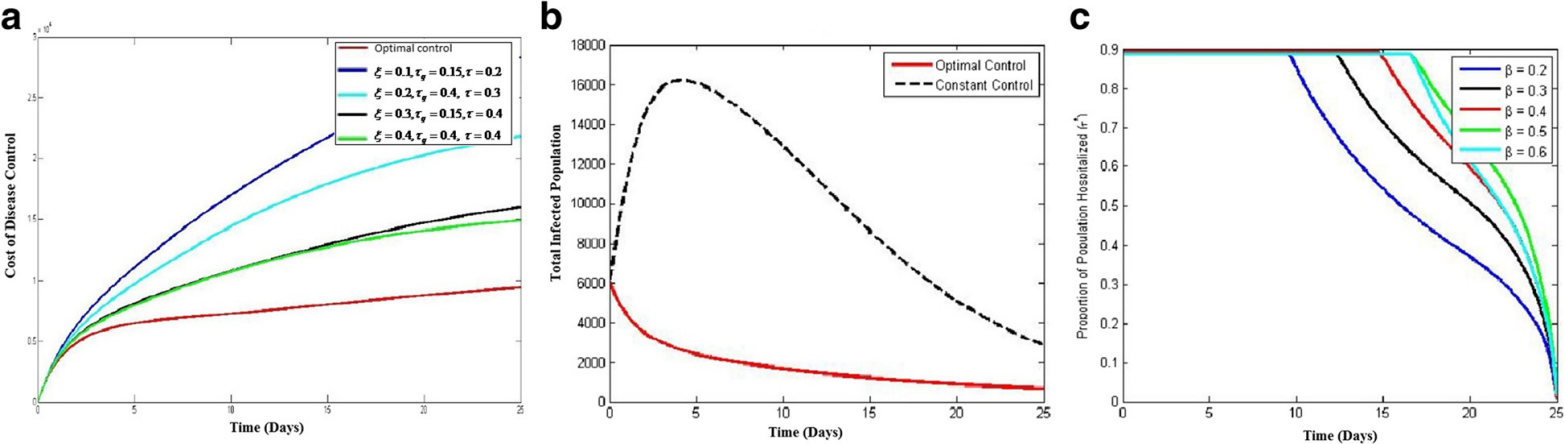
Optimal control case of hospitalization and quarantine

Figure 5a shows the comparison between the optimal control path and constant control path of the cost of disease control. The upper limit of *ζ* in the optimality condition is taken to be 0.6. It is the plot of the numerical solution of the cost functional *J*, mentioned in the analysis section (given in the Appendix). The figure clearly depicts a sound decrease in the cost total cost with the same efficacy level when compared to constant control factor. The effectiveness can be viewed from the difference between the cost values of each individual graphs with the optimal control path.

Panel (B) of Fig. 5 shows the comparison between the population of infected individuals using constant control and optimal control. The upper limit of *ζ* in the optimality condition is taken to be 0.6. It is the plot of the numerical solution of the infected population compartment, mentioned in the analysis section. The figure clearly depicts a sound decrease in the number of infected individuals when hospitalization and quarantine is applied optimally rather than constantly; the efficacy is evident from the results of the graph. The effectiveness can be viewed from the difference between the peaks of the two graphs.

Finally, in panel (C), the solution of the control variables for optimal path are depicted. Observe that as the values of *β* increase, the graphs translate horizontally to right. This is important because it yields the optimum rate of hospitalization at a given time, resulting in minimizing the overall cost. These graphs are the solutions to the cost functionals mentioned in the equation of *J* in analysis portion.

#### Hospitalization and vaccination

**Fig. 6:**
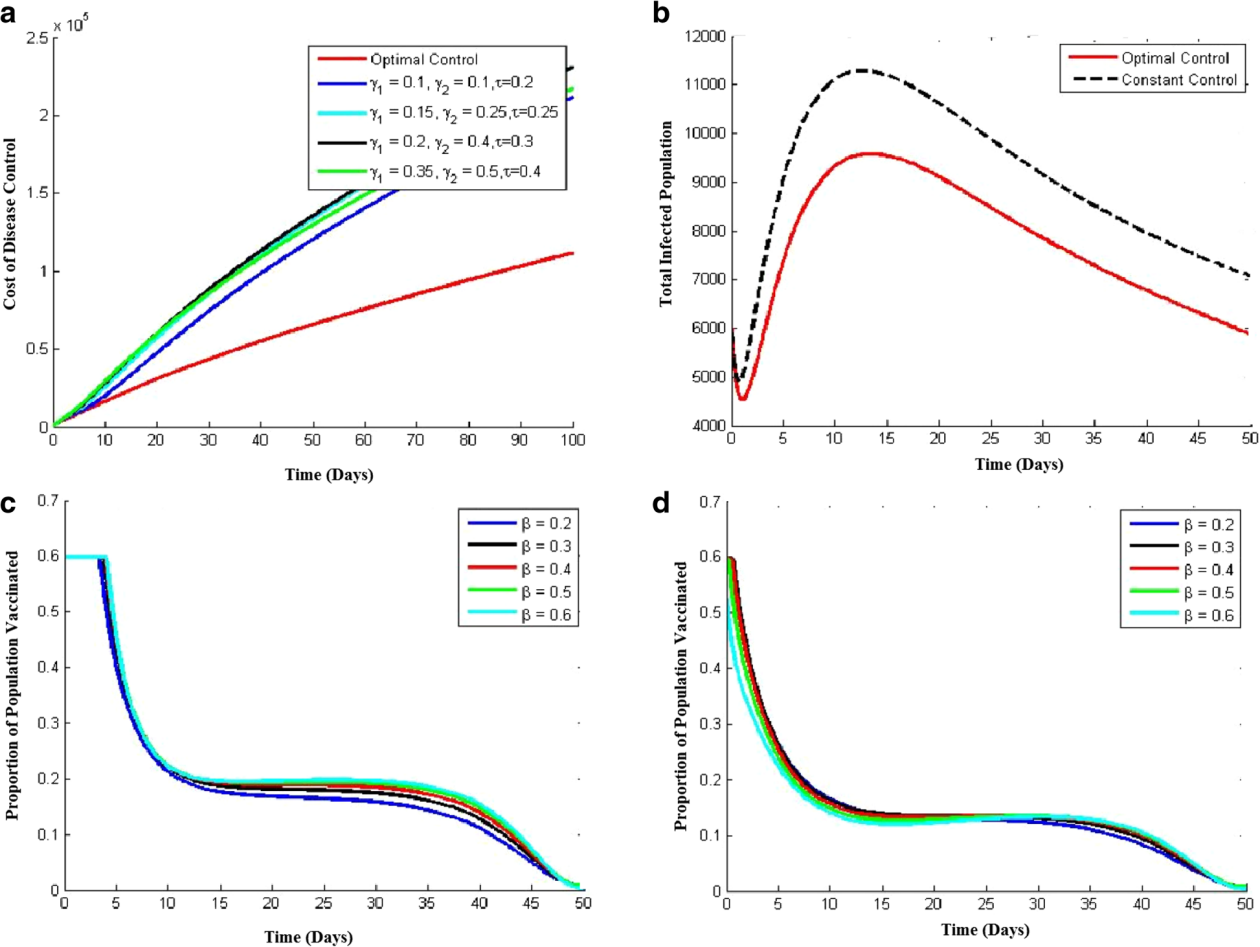
Optimal control case of hospitalization and vaccination

Simulation results of this case are depicted in Fig. 6. Panel (A) of the figure shows the comparison between the optimal control path and constant control path of the cost of disease control (see Appendix for the model equations). The upper limit of *ζ* in the optimality condition is taken to be 0.6. The figure clearly depicts a sound decrease in the total cost with the same efficacy level when compared to constant control factor. The effectiveness can be viewed from the difference between the cost values of each individual graphs with the optimal control path. The different values of constants of variables *γ*_1_, *γ*_2_ and *τ* are provided for making effective comparisons.

The comparison between the population of infected individuals using constant control and optimal control is depicted in Panel (B) of Fig. 6. The upper limit of *ζ* in the optimality condition is again taken to be 0.6. The figure clearly shows a sound decrease in the number of infected individuals when hospitalization and vaccination is applied optimally rather than constantly; the efficacy is evident from the results of the graph. The effectiveness can be viewed from the difference between the peaks of the two graphs.

In panel (C), the solution of the control variables for optimal path are shown. These results are important because they yield the optimum rate of hospitalization at a given time, resulting in minimizing the overall cost. These graphs are the solutions to the cost functionals mentioned in the equation of *J* in the analysis portion for the hospitalization variable (See Appendix) and for the case of vaccination in the “Low-risked susceptible”. It is clear that these functions do not vary a lot when the value of *β* is changed but there is a slight increase whenever *β* encounters a positive change. It can also be viewed that the optimality conditions being applied very early in the solution as the graphs dips down from 0.6, the upper bound, and starts giving lower values for the remaining part of time. Finally, similar results are obtained for the case of vaccination in the “High-risked susceptible individuals”. as shown in panel (D). Further, if we compare these results with the “Low-risked susceptible”, it can be viewed that the vaccination function of high risked individuals is controlled optimally better than that of low risked individuals, and this is what was desired as a higher portion of high risked individuals are to be vaccinated in a given time due to higher risks.

## Conclusion

In this paper we presented deterministic ordinary differential equations models to understand the Ebola virus disease dynamics. Our basic model incorporates a very important factor of individuals with high risk level with exposure to disease than the general population in the affected areas. These include front line health care workers, family members of EVD patients and Individuals involved in burial of deceased EVD patients. We perform mathematical analysis and computational simulations to suggest the possible methods to avoid and effectively control Ebola disease.

In the first part of the paper, we looked at the effect of interventions in order to control the outbreak. Our analysis suggests that in order for *R*_0_ to be less than one, the transmission rate of isolated individuals should be less than one-fourth of that for non-isolated ones. This means that strict protocols should be followed at treatment facilities. Further analysis of the model also leads to the conclusion that the fraction of high-risk individuals has to be controlled and must be brought to a significantly lower level in order to bring *R*_0_ less than one to effectively control the outbreak.

Sensitivity analysis was performed to ascertain the relative importance of various parameters used in our model. This would help epidemiologists and public health officials to focus on the more important parameters in formulating a disease control policy. Our analysis led to the observations that two parameters *θ*_*H*_ and *ψ*_*H*_ have significant contribution to the disease dynamics and need to be calculated precisely for an accurate outcome.

Second part of the paper was devoted to optimal control analysis and we employed Pontryagin’s Maximal principle and suggest the optimal strategies for controlling the disease. In our analysis we assumed a quadratic cost function due to the obvious non linearity of the cost, as briefly discussed in the optimal control section, and the fact that convexity of the function allows one to apply established results from optimal control theory, as has been done in similar work in the literature. Since recently, a new vaccination was found to be very effective for the disease treatment [8], we introduced two variations in our original model with “*Hospitalization and Quarantine*” and second with “*Hospitalization and Vaccination*”. Our analysis suggested that an optimal control (rather than a high constant control) is preferable, where the quarantining rate of infected individuals is a function of time. That is, the proportion that is quarantined optimally with respect to time has a higher favorable impact (as compared to implementing a high but constant quarantine rate) in keeping the cost of disease control low. Similarly, in the second variation, our analysis successfully demonstrate the effectiveness of vaccination to control the outbreak.

It must be noted that optimal strategies while theoretically justified may be hard to implement in practice. As our analysis showed, we need high levels of medication and hospitalization at the beginning of an epidemic. This is problematic due to two reasons: first, it may not be feasible due to the availability and costs incurred in medicating and/or hospitalizing a significant fraction of the total susceptible population; second, the control strategies need to be at a maximum at the beginning of the epidemic. This is hard to implement as there is bound to be a time lag between the onset of an epidemic and when the public health authorities realize it is in progress, moreover even after such a realization it would take time to implement the strategies. But in spite of all the hurdles, these optimal strategies may be used by public health authorities to determine quasi-optimal strategies they might want to adopt and the relative impact of such strategies on the epidemic.

## Appendix: optimal control analysis

### Case 1: hospitalization and quarantine

We use Pontrygain’s Maximum Principle on our model system (4), and the Hamiltonian is given by

$${} \begin{aligned} &H^{*}= I +Q+ \frac{1}{2} W_{1}\xi^{2}+\frac{1}{2} W_{2}{\tau_{Q}^{2}}(t)+\frac{1}{2} W_{3}\tau^{2}(t)\\ &+ \phi_{1}\left[\!\Pi(1-p)-\lambda S_{L}-\mu S_{L}\right] +\phi_{2}\left[\!\Pi p-\psi_{_{H}}\lambda S_{H} -\mu S_{H}\right]\\ &+\phi_{3}\left[\lambda (S_{L}+ \psi_{_{H}} S_{H})-(\alpha+\mu)E-\xi E\right]\\ & +\phi_{4}\left[\xi E -(\tau_{_{Q}} +\mu)Q\right]\\ &+\phi_{5}\left[\alpha E - (\tau +\theta_{I} +\delta_{I} +\mu)I\right] +\phi_{6}\\ &~\qquad\left[\tau I +\tau_{_{Q}}Q -(\theta_{H} +\delta_{H} +\mu)H\right]\\ &+\phi_{7}\left[\theta_{I} I +\theta_{H} H-\mu R\right] \end{aligned} $$

**Claim** There exist unique optimal controls *ξ*^∗^(*t*), $\tau _{Q}^{*}(t)$ and *τ*^∗^(*t*) which minimize *J* over **U**. Also there exists an adjoint system of *ϕ*_*i*_’s such that the optimal treatment control is characterized as

13 $$\begin{array}{@{}rcl@{}} \xi^{*}(t)={min}\left[\zeta_{1}, {max}\left(0, \frac{E(\phi_{3}-\phi_{4})}{W_{1}}\right)\right] \end{array} $$

14 $$\begin{array}{@{}rcl@{}} \tau_{Q}^{*}(t)={min}\left[\zeta_{2}, {max}\left(0, \frac{Q (\phi_{4}-\phi_{6})}{W_{2}}\right)\right] \end{array} $$

15 $$\begin{array}{@{}rcl@{}} \tau^{*}(t)={min}\left[\zeta_{3}, {max}\left(0, \frac{I (\phi_{4}-\phi_{5})}{W_{3}}\right)\right] \end{array} $$

Applying the first condition of the Pontrygain’s Maximum Principle we get the adjoint system.

16 $$\begin{array}{@{}rcl@{}} \frac{\partial {H^{*}}}{\partial{\xi}} =& 0\Rightarrow \xi = \left(\frac{E\left(\phi_{3}-\phi_{4}\right)}{W_{1}}\right) \end{array} $$

17 $$\begin{array}{@{}rcl@{}} \frac{\partial {H^{*}}}{\partial{\tau_{Q}}} =& 0\Rightarrow \tau_{Q} = \left(\frac{Q \left(\phi_{4}-\phi_{6}\right)}{W_{2}}\right) \end{array} $$

18 $$\begin{array}{@{}rcl@{}} \frac{\partial {H^{*}}}{\partial{\tau}} =& 0\Rightarrow \tau = \left(\frac{I (\phi_{4}-\phi_{5})}{W_{3}}\right) \end{array} $$

Similarly applying the second condition

19 $$\begin{array}{@{}rcl@{}} \frac{d\phi_{1}}{dt}&= -\frac{\partial H^{*}}{\partial S_{L}} \end{array} $$

20 $$\begin{array}{@{}rcl@{}} \frac{d\phi_{2}}{dt}&= -\frac{\partial H^{*}}{\partial S_{H}} \end{array} $$

21 $$\begin{array}{@{}rcl@{}} &\quad\vdots \quad\vdots \end{array} $$

22 $$\begin{array}{@{}rcl@{}} \frac{d\phi_{7}}{dt}&=- \frac{\partial H^{*}}{\partial R} \end{array} $$

The adjoint system is given as

23 $$\begin{array}{@{}rcl@{}} \frac{d\phi_{1}}{dt}&=\left(\lambda+\mu \right)\phi_{1} -\lambda \phi_{2} \end{array} $$

24 $$\begin{array}{@{}rcl@{}} \frac{d\phi_{2}}{dt}&=\psi_{H} \lambda \phi_{2} + \mu \phi_{2} - \psi_{H} \lambda \phi_{3} \end{array} $$

25 $$\begin{array}{@{}rcl@{}} \frac{d\phi_{3}}{dt}&= (\alpha +\mu +\xi) \phi_{3} -\xi \phi_{4} -\xi \phi_{5} \end{array} $$

26 $$\begin{array}{@{}rcl@{}} \frac{d\phi_{4}}{dt}&=-1+(\tau_{Q}+ \mu) \phi_{4}-\tau_{Q} \phi_{6} \end{array} $$

27 $$\begin{array}{@{}rcl@{}} \frac{d\phi_{5}}{dt}&=-1 + \left(\frac{\beta}{N}\right) S_{L} \phi_{1} +\left(\frac{\beta}{N}\right) S_{H} \psi_{H} \phi_{2}-\left(\frac{\beta}{N}\right) \\ &\times(S_{L}+S_{H}\psi_{H}) \phi_{3} \end{array} $$

28 $$\begin{array}{@{}rcl@{}} &+(\tau \theta_{I} +\delta_{I} +\mu) \phi_{5} -\tau \phi_{6}-\theta_{I} \phi_{7} \end{array} $$

29 $$\begin{array}{@{}rcl@{}} \frac{d\phi_{6}}{dt}&= \left(\frac{\beta\eta}{N}\right) S_{L} \phi_{1} +\left(\frac{\beta\eta}{N}\right) S_{H} \psi_{H} \phi_{2}-\left(\frac{\beta\eta}{N}\right)\\ &\times (S_{L}+S_{H}\psi_{H}) \phi_{3} \end{array} $$

30 $$\begin{array}{@{}rcl@{}} &+(\theta_{H} +\delta_{H} +\mu) \phi_{6} -\theta_{H} \phi_{7} \end{array} $$

31 $$\begin{array}{@{}rcl@{}} \frac{d\phi_{7}}{dt}&=\mu \phi_{7} \end{array} $$

The above adjoint system also satisfies the transversality condition, {*ϕ*_*i*_(*T*)=0:*i*=1,2,⋯,7}.

### Case 2: hospitalization and vaccination

Again using Pontrygain’s Maximum Principle on system (5), the Hamiltonian is given as

32 $$\begin{array}{@{}rcl@{}}{} H^{*}&= I + \frac{1}{2} W_{1}{\gamma_{1}^{2}}+\frac{1}{2} W_{2}{\gamma_{2}^{2}}(t)+\frac{1}{2} W_{3}\tau^{2}(t) \end{array} $$

33 $$\begin{array}{@{}rcl@{}} &+ \phi_{1}\left[\Pi(1-p)-\lambda S_{L}-\gamma_{1} S_{L}-\mu S_{L}\right] \end{array} $$

34 $$\begin{array}{@{}rcl@{}} & +\phi_{2}\left[\Pi p-\psi_{_{H}}\lambda S_{H} -\gamma_{2} S_{H}-\mu S_{H}\right] \end{array} $$

35 $$\begin{array}{@{}rcl@{}} &+\phi_{3}\left[\gamma_{1} S_{L} +\gamma_{2} S_{H} -\lambda(1-\epsilon)V-\mu V\right] \end{array} $$

36 $$\begin{array}{@{}rcl@{}} &+\phi_{4}\left[\lambda (S_{L}+ \psi_{_{H}} S_{H})+\lambda(1-\epsilon)V-(\alpha+\mu)E\right] \end{array} $$

37 $$\begin{array}{@{}rcl@{}} &+\phi_{5}\left[\alpha E - (\tau +\theta_{I} +\delta_{I} +\mu)I\right] \end{array} $$

38 $$\begin{array}{@{}rcl@{}} &+\phi_{6}\left[\tau I -(\theta_{H} +\delta_{H} +\mu)H\right] \end{array} $$

39 $$\begin{array}{@{}rcl@{}} &+\phi_{7}\left[\theta_{I} I +\theta_{H} H-\mu R\right] \end{array} $$

**Claim** There exist unique optimal controls $\gamma _{1}^{*}(t)$, $\gamma _{2}^{*}(t)$ and *τ*^∗^(*t*) which minimize *J* over **U**. Also there exists an adjoint system of *ϕ*_*i*_’s such that the optimal treatment control is characterized as

40 $$\begin{array}{@{}rcl@{}} \gamma_{1}^{*}(t)&={min}\left[\zeta_{1}, {max}\left(0, \frac{S_{L}(\phi_{1}-\phi_{3})}{W_{1}}\right)\right] \end{array} $$

41 $$\begin{array}{@{}rcl@{}} \gamma_{2}^{*}(t)&={min}\left[\zeta_{2}, {max}\left(0, \frac{S_{H} (\phi_{2}-\phi_{3})}{W_{2}}\right)\right] \end{array} $$

42 $$\begin{array}{@{}rcl@{}} \tau^{*}(t)&={min}\left[\zeta_{3}, {max}\left(0, \frac{I (\phi_{5}-\phi_{6})}{W_{3}}\right)\right] \end{array} $$

Applying the first condition of the Pontrygain’s Maximum Principle

43 $$\begin{array}{@{}rcl@{}} \frac{\partial {H^{*}}}{\partial{\gamma_{1}}} =& 0\Rightarrow \gamma_{1} = \left(\frac{S_{L}(\phi_{1}-\phi_{3})}{W_{1}}\right) \end{array} $$

44 $$\begin{array}{@{}rcl@{}} \frac{\partial {H^{*}}}{\partial{\gamma_{2}}} =& 0\Rightarrow \gamma_{2} = \left(\frac{S_{H} (\phi_{2}-\phi_{3})}{W_{2}}\right) \end{array} $$

45 $$\begin{array}{@{}rcl@{}} \frac{\partial {H^{*}}}{\partial{\tau}} =& 0\Rightarrow \tau = \left(\frac{I (\phi_{5}-\phi_{6})}{W_{3}}\right) \end{array} $$

Similarly applying the second condition

46 $$\begin{array}{@{}rcl@{}} \frac{d\phi_{1}}{dt}&= -\frac{\partial H^{*}}{\partial S_{L}} \end{array} $$

47 $$\begin{array}{@{}rcl@{}} \frac{d\phi_{2}}{dt}&= -\frac{\partial H^{*}}{\partial S_{H}} \end{array} $$

48 $$\begin{array}{@{}rcl@{}} &\quad\vdots\quad \vdots \end{array} $$

49 $$\begin{array}{@{}rcl@{}} \frac{d\phi_{7}}{dt}&=- \frac{\partial H^{*}}{\partial R} \end{array} $$

The adjoint system is given as

50 $$\begin{array}{@{}rcl@{}} \frac{d\phi_{1}}{dt}&=\left(\lambda+\mu+\gamma_{1} \right)\phi_{1} -\lambda \phi_{4}-\gamma_{1} \phi_{3} \end{array} $$

51 $$\begin{array}{@{}rcl@{}} \frac{d\phi_{2}}{dt}&=\left(\psi_{H} \lambda+\gamma_{2} +\mu \right) \phi_{2} - \gamma_{2} \phi_{2} - \lambda \phi_{4} \end{array} $$

52 $$\begin{array}{@{}rcl@{}} \frac{d\phi_{3}}{dt}&= \left(\lambda(1-\epsilon) +\mu \right) \phi_{3} -\left(\lambda(1-\epsilon)\right) \phi_{4} \end{array} $$

53 $$\begin{array}{@{}rcl@{}} \frac{d\phi_{4}}{dt}&=(\alpha+ \mu) \phi_{4}-\alpha\phi_{5} \end{array} $$

54 $$\begin{array}{@{}rcl@{}} \frac{d\phi_{5}}{dt}&=-1 + \left(\frac{\beta}{N}\right) S_{L} \phi_{1} +\left(\frac{\beta}{N}\right) S_{H} \psi_{H} \phi_{2}+\left(\frac{\beta}{N}\right)\\ &\times(1-\epsilon)V \phi_{3} \end{array} $$

55 $$\begin{array}{@{}rcl@{}} &-\left[\left(\frac{\beta}{N}\right)(S_{L}+S_{H}\psi_{H})+\left(\frac{\beta}{N}\right)(1-\epsilon)V\right] \phi_{4} \end{array} $$

56 $$\begin{array}{@{}rcl@{}} &+(\tau+ \theta_{I} +\delta_{I} +\mu) \phi_{5} -\tau \phi_{6}-\theta_{I} \phi_{7} \end{array} $$

57 $$\begin{array}{@{}rcl@{}} \frac{d\phi_{6}}{dt}&= \left(\frac{\beta\eta}{N}\right) S_{L} \phi_{1} +\left(\frac{\beta\eta}{N}\right) S_{H} \psi_{H} \phi_{2} +\left(\frac{\beta\eta}{N}\right)\\ &\times(1-\epsilon)V \phi_{3} \end{array} $$

58 $$\begin{array}{@{}rcl@{}} &-\left[\left(\frac{\beta\eta}{N}\right)(S_{L}+S_{H}\psi_{H})+\left(\frac{\beta\eta}{N}\right) (1-\epsilon)V \right] \phi_{4} \end{array} $$

59 $$\begin{array}{@{}rcl@{}} &+(\theta_{H} +\delta_{H} +\mu) \phi_{6} -\theta_{H} \phi_{7} \end{array} $$

60 $$\begin{array}{@{}rcl@{}} \frac{d\phi_{7}}{dt}&=\mu \phi_{7} \end{array} $$

The above adjoint system also satisfies the transversality condition, {*ϕ*_*i*_(*T*)=0:*i*=1,2,⋯,7}.

## Additional file

Additional file 1 Please see Additional file 1 for translations of the abstract into the five official working languages of the United Nations. (PDF 378 kb)
