# Supplementary material for: Optimal control analysis of Ebola disease with control strategies of quarantine and vaccination
Source: Infect Dis Poverty. 2016 Jul 13;5:72. doi: 10.1186/s40249-016-0161-6 (PMC4942907; doi:10.1186/s40249-016-0161-6)
Supplement: Additional file 1 — Please see Additional file 1 for translations of the abstract into the five official working languages of the United Nations. (PDF 378 kb) [file 40249_2016_161_MOESM1_ESM.pdf]

Translation of the abstract into the five official working languages of the United Nations

حليل التحكم الأمثل لمرض إيبولا مع استراتيجيات التحكم في الحجر الصحي والتطعيم

الدر أحمد محمد، عثمان محمد خان عدنان وعمران مدثر

ملخص

خلفية: وباء الإيبولا الذي ظهر عام 2014 هو الأكبر في التاريخ، وأحدث تأثيرا في عدة دول في غرب أفريقيا. وقد لوحظت بعض حالات معزولة أيضا في مناطق أخرى من العالم.

الطريقة: في هذه الورقة، نقدم نموذجا تحديديا من نوع SEIR (الأفراد عرضة للإصابة، والأفراد المعرضين والأفراد مصدر العدوى، والأفراد المتعافين) مع مكونات استشفاء، وحجر صحي وتطعيم إضافية من أجل فهم ديناميات المرض. استراتيجيات التحكم الأمثل، سواء في حالة دخول المستشفى (مع وبدون الحجر الصحي) والتطعيم يتم استخدامها للتنبؤ بالنتائج المحتملة في المستقبل من حيث استخدام الموارد لمكافحة الأمراض وفعالية التطعيم على السكان المرضى. وعلاوة على ذلك، وبمساعدة تحليل عدم اليقين والحساسية حددنا المعايير الأكثر حساسية والتي تسهم بشكل فعال في تغيير ديناميات المرض. وقد أجرينا التحليل الرياضي مع المحاكاة العددية واستراتيجيات التحكم الأمثل على نماذج فيروس الإيبولا.

النتائج: استخدمنا أدوات نظام الديناميكية مع المحاكاة العددية واستراتيجيات التحكم الأمثل في نماذج فيروس الإيبولا الخاصة بنا. النموذج الأصلي، والذي سمح بانتقال فيروس الإيبولا عن طريق الاتصال البشري، امتد ليشمل التطعيم غير الكامل والحجر الصحي. بعد التحليل النوعي لجميع الأشكال الثلاثة لنموذج الإيبولا صيغت التقنيات الرقمية، وذلك باستخدام MATLAB كمنصة وتم تحليلها بالتفصيل. وتدعم نتائج المحاكاة التي توصلنا لها الفرضيات الواردة في القسم النوعي.

الخلاصة: يتضمن نموذجنا عنصرا هاما من الأفراد المعرضين لمستوى مخاطر عالية للتعرض للمرض، مثل العاملين في الرعاية الصحية، وأفراد أسرة المرضى المصابين بمرض فيروس الإيبولا EVD والأفراد المشاركين في دفن المرضى المتوفين المصابين بمرض فيروس الإيبولا، عن عامة السكان في المناطق المتأثرة بالمرض. ويشير تحليلنا أنه من أجل أن يكون R0 (أي عدد الاستنساخ الأساسي) أقل من واحد، وهذا وهو الشرط الأساسي للقضاء المرض، يجب أن يكون معدل نقل الأفراد المعزولين أقل من ربع هؤلاء الموجودين في المناطق غير المعزولة. ويتوقع تحليلنا أيضا، أننا بحاجة إلى مستويات عالية من الدواء والاستشفاء عند بدء حدوث الوباء. وعلاوة على ذلك، يقترح تحليل التحكم الأمثل للنموذج استراتيجيات المكافحة التي يمكن اعتمادها من قبل سلطات الصحة العامة من أجل الحد من تأثير الأوبئة مثل الإيبولا.

Translated from English version into Arabic by Mahmoud Sami, through

TRANSLATORS
WITHOUT BORDERS

埃博拉病毒病的最优控制策略分析：隔离和疫苗接种控制策略

Dur-e-Ahmad Muhammad, Usman Muhammad, Khan Adnan and Imran Mudassar

摘要

引言：2014 年埃博拉病毒病流行是历史上规模最大的，影响了西非众多国家。此外，在世界其他区域还有一些孤立的病例。

方法：通过确定性 SEIR 类型模型结合住院、隔离和疫苗接种因素研究埃博拉疾病的动态变化。在住院治疗(有/无隔离)和接种疫苗的情况下，疾病控制中的资源利用和患病人群接种疫苗的有效性均采用最优控制策略预测未来可能的结果。在不确定性和敏感性分析的帮助下，确定了最敏感的能有效地改变疾病动力学的参数。并对数值模拟和埃博拉病毒模型的最优控制策略进行了数学分析。

结果：采用数值模拟的动力学系统工具和最优控制策略研究埃博拉病毒模型。初始模型将允许通过人类接触导致埃博拉病毒的传播，扩展至非严格的疫苗接种和隔离。对所有 3 种形式的埃博拉病毒模型进行定性分析后，以 MATLAB 为平台的数值技术计算并进行详细分析，本研究的模拟结果支持定性部分的结论。
结论：我们的模型包含了暴露于疾病的风险水平高的个体的重要组分，如前线医护人员、埃博拉病毒病 EVD 患者的家庭成员和参与埋葬去世 EVD 患者的个人，而不是受影响的地区的一般人群。分析表明，为了使 R0（如基本再生数）小于 1，该指标是消除疾病的最基本要求，单个个体的传输速率应该小于非独立个体的 1/4。同时，我们预测在疾病流行伊始需要高水平的药物治疗和住院治疗。对模型的最优控制分析表明，一些控制策略可被公共卫生部门所采用以控制诸如埃博拉流行所造成的影响。

Translated from English version into Chinese by Xin-Yu Feng, edited by Pin Yang, through

CHINESE CENTER FOR DISEASE CONTROL AND PREVENTION
NATIONAL INSTITUTE OF PARASITIC DISEASES

Analyse de la lutte optimale contre le virus Ebola par des stratégies de quarantaine et de vaccination

Dur-e-Ahmad Muhammad, Usman Muhammad, Khan Adnan et Imran Mudassar

Résumé

Contexte : L’épidémie d’Ebola de 2014 est la plus importante du monde et a affecté de très nombreux pays d’Afrique occidentale. Certains cas isolés ont aussi été observés dans d’autres régions du monde.

Méthode : Cet article nous permet de présenter un modèle de type SEIR déterministe tenant compte de composants supplémentaires d’hospitalisation, de quarantaine et de vaccination afin de comprendre les dynamiques d’une maladie. Des stratégies de lutte optimale, à la fois en cas d’hospitalisation (avec et sans quarantaine) et de vaccination sont utilisées afin de prédire le futur résultat potentiel en termes d’exploitation des ressources pour la lutte contre la maladie ainsi que l’efficacité de la vaccination sur des populations malades. De plus, nous nous sommes aidés d’une analyse d’incertitude et de sensibilité pour identifier les paramètres les plus sensibles contribuant effectivement à un changement dans les dynamiques d’une maladie. Nous avons réalisé une analyse mathématique sur la base de simulations numériques et de stratégies de lutte optimale sur des modèles de virus Ebola.

Résultats : Nous avons utilisé des outils de système dynamiques reposant sur des simulations numériques et des stratégies de lutte optimale sur nos modèles du virus Ebola. Le modèle original qui a permis la transmission du virus Ebola par contact humain a été étendu pour inclure une vaccination et une quarantaine imparfaites. Suite à l’analyse qualitative de l’ensemble des trois formes du modèle Ebola, des techniques numériques, reposant sur le logiciel MATLAB à titre de plate-forme, ont été élaborées et analysées en détail. Les résultats de notre simulation appuient les exigences communiquées en matière de qualité.

Conclusion : Notre modèle comprend un important composant que sont les individus à risque élevé et exposés à la maladie, tels que les travailleurs de la santé de première ligne, les membres de la famille de patients atteints du virus Ebola et les individus affectés à l’enterrement de patients atteints du virus Ebola décédés, plutôt que d’intégrer la population générale dans les zones affectées. Notre analyse suggère que pour que le R0 (c’est-à-dire le taux de reproduction de base) soit inférieur à un, ce qui constitue l’exigence de base pour l’élimination de la maladie, le taux de transmission d’individus isolés doit être inférieur au quart de celui des individus non isolés. Notre analyse prédit aussi que nous avons besoin de niveaux élevés de médication et d’hospitalisation au début d’une épidémie. De plus, une analyse de la lutte optimale du modèle suggère les stratégies de lutte qui peuvent être adoptées par les autorités de santé publique afin de réduire l’impact d’épidémies comme Ebola.

Translated from English version into French by eric ragu, through

Анализ оптимального контроля лихорадки Эбола со стратегиями контроля карантина и вакцинации

Dur-e-Ahmad Muhammad, Usman Muhammad, Khan Adnan и Imran Mudassar

Аннотация

Краткая информация: Эпидемия Эбола 2014 года стала крупнейшей в истории, затронув несколько стран в Западной Африке. Некоторые единичные случаи наблюдались и в других регионах мира.

Метод: В этой статье мы вводим детерминированную модель типа SEIR с дополнительными компонентами госпитализацию, карантина и вакцинации для того, чтобы понять динамику заболевания. Оптимальные стратегии контроля, как в случае госпитализации (с карантином и без него), так и вакцинации, используются для прогнозирования возможных будущих результатов с точки зрения использования ресурсов для борьбы с болезнями и эффективности вакцинации среди популяций пациентов. Кроме того, с помощью анализа неопределенности и чувствительности мы также определили наиболее чувствительные параметры, которые эффективно способствуют изменениям динамики заболевания. Мы провели математический анализ с результатами численного моделирования и оптимальных стратегий управления на моделях вируса Эбола.

Результаты: Мы использовали инструменты динамической системы с численным моделированием и оптимальными стратегиями контроля на наших моделях вируса Эбола. Оригинальная модель, которая предусматривала передачу вируса Эбола через контакт с человеком, была расширена для включения несовершенных вакцинации и карантина. После качественного анализа всех трех форм модели Эбола, численные методы, с использованием MATLAB в качестве платформы, были сформулированы и проанализированы в деталях. Наши результаты моделирования свидетельствуют в пользу заявлений, сделанных в качественном разделе.

Заключение: Наша модель включает в себя такие важные компоненты, как лица с высоким уровнем риска заболевания, такие как медицинские работники, непосредственно контактирующие с больными, члены семей больных геморрагической лихорадкой Эбола, и лица, участвующие в захоронении умерших больных геморрагической лихорадкой Эбола, а не общее население пострадавших районов. Наш анализ показывает, что для того, чтобы R0 (то есть, основное число воспроизведения) было меньше единицы, что является основным требованием для ликвидации заболевания, скорость передачи от изолированных лиц должна быть менее одной четверти скорости передачи от неизолированных лиц. Наш анализ также предсказывает, что нам требуется значительное количество медикаментов и высокие уровни госпитализации в начале эпидемии. Кроме того, анализ оптимального контроля модели предполагает стратегии контроля, которые могут быть приняты органами здравоохранения с целью снижения последствий таких эпидемий, как Эбола.

Translated from English version into Russian by Oksana Weiss, through

Análisis del control óptimo de la enfermedad del ébola con estrategias de control para cuarentenas y vacunación

Dur-e-Ahmad Muhammad, Usman Muhammad, Khan Adnan e Imran Mudassar

Resumen

Antecedentes: La epidemia de ébola del año 2014 es la más grande en la historia, llegando a afectar a varios países en África Occidental. También se observaron algunos casos aislados en otras regiones del mundo.

Método: En este estudio, introducimos un modelo determinístico tipo SEIR con componentes adicionales de hospitalización, cuarentena y vacunación para poder comprender la dinámica de la enfermedad. Se utilizaron estrategias de control óptimo, tanto en el caso de hospitalización (con o sin cuarentena) como en el de vacunación para predecir los posibles resultados en el futuro en términos de utilización de recursos para el control de la enfermedad y la eficacia de la vacunación en poblaciones enfermas. Más aún, con la ayuda del análisis de incertidumbre y sensibilidad también hemos podido identificar los parámetros más sensibles que contribuyen eficazmente al cambio en la dinámica de la enfermedad. Hemos llevado a cabo análisis matemáticos con simulaciones numéricas y estrategias de control óptimo en los modelos de virus del ébola .

Resultados: En nuestros modelos de virus del ébola utilizamos herramientas de sistema dinámicas con simulaciones numéricas y estrategias de control óptimo. El modelo original, que permitió la transmisión del virus del ébola vía contacto humano, se extendió para incluir vacunas imperfectas y cuarentena. Luego del análisis cualitativo de las tres formas del modelo del ébola, se formularon y analizaron en detalle técnicas numéricas mediante el uso de MATLAB como plataforma. Nuestros resultados de la simulación apoyaron las afirmaciones que se hicieron en la sección cualitativa.

Conclusión: Nuestro modelo incorpora un componente significativo de personas con un nivel de riesgo alto de exposición a la enfermedad, como los trabajadores de salud de primera línea, familiares de pacientes con EVE y personas involucradas en el entierro de pacientes con EVE fallecidos, en lugar de la población general en las zonas afectadas. Nuestro análisis sugiere que para que R0 (es decir, el número de reproducción básico) sea menor que uno, que es el requisito básico para la eliminación de la enfermedad, el índice de transmisión del ébola de personas aisladas debería ser de menos de un cuarto que el de aquellas personas no aisladas. Nuestro análisis también predice que necesitamos altos niveles de medicamentos y hospitalización al inicio de cualquier epidemia. Es más, el análisis de control óptimo del modelo sugiere las estrategias de control que podrían llegar a adoptar las autoridades de salud pública para disminuir el impacto de epidemias como la del ébola.

Translated from English version into Spanish by Maria Alejandra Aguada, through
